# Recent Progress in Bioconjugation Strategies for Liposome-Mediated Drug Delivery

**DOI:** 10.3390/molecules25235672

**Published:** 2020-12-01

**Authors:** Bethany Almeida, Okhil K. Nag, Katherine E. Rogers, James B. Delehanty

**Affiliations:** 1 American Society for Engineering Education, Washington, DC 20036, USA; bethany.almeida.ctr@nrl.navy.mil; 2 Center for Bio/Molecular Science and Engineering, Code 6900, U.S. Naval Research Laboratory, Washington, DC 20375, USA; okhil.nag@nrl.navy.mil (O.K.N.); katherine.rogers@nrl.navy.mil (K.E.R.); 3 Fischell Department of Bioengineering, 2330 Kim Engineering Building, University of Maryland, College Park, MD 20742, USA

**Keywords:** liposome, bioconjugation, drug delivery, targeting

## Abstract

In nanoparticle (NP)-mediated drug delivery, liposomes are the most widely used drug carrier, and the only NP system currently approved by the FDA for clinical use, owing to their advantageous physicochemical properties and excellent biocompatibility. Recent advances in liposome technology have been focused on bioconjugation strategies to improve drug loading, targeting, and overall efficacy. In this review, we highlight recent literature reports (covering the last five years) focused on bioconjugation strategies for the enhancement of liposome-mediated drug delivery. These advances encompass the improvement of drug loading/incorporation and the specific targeting of liposomes to the site of interest/drug action. We conclude with a section highlighting the role of bioconjugation strategies in liposome systems currently being evaluated for clinical use and a forward-looking discussion of the field of liposomal drug delivery.

## 1. Introduction

Since their first published description in 1964 [1], interest in liposomes as drug carriers and delivery agents has grown exponentially, and they have become the most prominent nanoparticle (NP) drug delivery system approved for use in the clinic. This is due in a large part to: 1—their facile fabrication methods, 2—their ability to encapsulate a wide variety of drugs and molecules (regardless of hydrophobicity, charge, size, and other physicochemical properties), and 3—their biocompatibility. Since the FDA approval of Doxil^®^ (a liposomal formulation of the anticancer drug, doxorubicin) in 1995 [2], more than a dozen liposomal drug delivery systems have been approved for clinical use with many more currently in the pipeline (see Section 5) [3,4,5].

**Figure 1:**
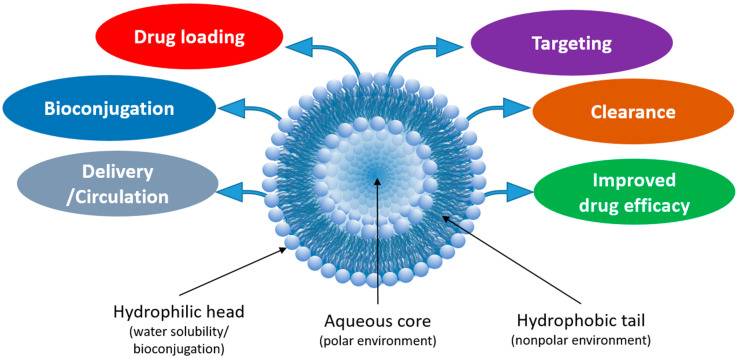
Structural features of liposomes and their advantageous features for improved drug delivery and therapeutic efficacy. Liposomes feature an aqueous core surrounded by a lipid bilayer, which creates an environment for loading hydrophilic or hydrophobic drugs, respectively. Functional groups on the lipid head enable conjugation to polymers, peptides, proteins, etc., for enhanced targeting, circulation, and overall drug efficacy.

Liposomes (NPs typically 100–500 nm in diameter) are fabricated from the self-assembly of phospholipids, which consist of a polar phosphate head group and hydrophobic lipid tails [6]. In aqueous environments, the hydrophobic tails self-orient, resulting in a spherical structure comprised of an aqueous core surrounded by a lipophilic bilayer membrane. Liposomes are both biocompatible and biodegradable (e.g., at certain pH and temperature), which can be controlled by modifying the lipid composition. Furthermore, liposomes are amenable to various modifications that improve their efficacy as drug delivery carriers (Figure 1). For example, “stealth” liposomes have been fabricated by the adsorption of a polyethylene glycol (PEG) layer on the liposome surface to “shield” the NP from renal clearance, thereby increasing circulation time [6,7]. The addition of PEG also increases the hydrophilicity of the liposome and the subsequent stability of the liposomes in aqueous storage. Drugs or other small molecules can be incorporated into the hydrophilic core or encapsulated within the hydrophobic bilayer during fabrication or a combination of both approaches can be used. For delivery to specific cell types/tissues, liposomes can be modified with proteins or small molecules to form targeted bioconjugates [8]. Due to the wide array of drugs and small molecules that can be incorporated within liposomes, liposomes can be used as combinational agents for simultaneous tissue/cellular imaging and drug delivery.

In this review, we highlight recent progress made in bioconjugation strategies for liposome-mediated drug delivery, with a particular emphasis on approaches for both drug incorporation and liposome targeting. To collect recent, relevant studies, we searched PubMed and related search engines for studies published within the last 5 years, using “liposome” as the main keyword; studies were organized by the bioconjugation method for both drug encapsulation and liposome targeting. Before we discuss the recent progress, it is first important to understand some of the essential design considerations in creating liposome–drug bioconjugates, which include controlled drug release, liposome targeting moieties, and liposome tracking. The first design consideration is how the drug cargo can be controllably released. By modifying the lipid composition, the liposome may be triggered to degrade in the presence of stimuli [9] such as enzymes, light, pH, temperature, or ultrasound or they may degrade passively, allowing the drug to slowly diffuse from the liposome. In this way, the rate of drug release can be controlled (i.e., zero-order release vs. first-order release and sustained vs. burst release). The concentration of drug loading also becomes an important consideration that can affect the drug release rate and overall efficacy. These factors should be designed based on the spatiotemporal control of drug delivery required for the particular application. For example, to treat a systemic infection, it may be useful to have a long-circulating, slow-release liposome, whereas for a tumor, it may be more useful to have a targeted liposome that quickly collects at the site of interest and only releases the drug when reaching the tumor.

The second critical design consideration is liposome targeting [6]. If the particular drug delivery application requires targeted liposome delivery, then one must consider the level of control over targeting that is most beneficial. Here it is important to factor in how specific the targeting should be (i.e., are only a specific subset of cells in a region meant to be targeted?). One must also consider the appropriate density of targeting ligands on the surface of the liposome to ensure optimal targeting without negatively affecting other liposome properties.

The final design consideration is in tracking—both in tracking of the liposome itself and monitoring drug release. For in vitro or in vivo applications, one must determine whether the liposome should be loaded with an imaging agent or if drug efficacy can be assessed without visualization. Proper design of the liposome is crucial for optimized drug delivery, and bioconjugation strategies may be employed to achieve this goal. In the sections that follow, we highlight current approaches that employ various bioconjugation strategies to optimize drug loading and release and liposome targeting. We also provide a brief synopsis of the current state of preclinical liposomal systems that are in phase I or phase II trials.

## 2. Strategies for Incorporation of Drugs into Liposomal Carriers

A major challenge in the formulation of liposome-based materials for drug delivery is the loading of the drug cargo(s) into/onto the liposomal carrier [10,11,12,13]. Poor drug loading efficiency not only wastes valuable drug molecules but also compromises therapeutic outcomes in experimental and clinical applications [14,15]. Unlike many other NPs, liposomes offer the advantage of having multiple different environments wherein the drug cargo can be interfaced. These include the hydrophilic aqueous core, the lipophilic phospholipid membrane bilayer, and the NP surface for direct drug conjugation/association [16,17]. These different environments offer varying degrees of drug loading capacity, release kinetics, protection against innate drug toxicity, tailored pharmacokinetic profiles, and overall therapeutic efficacy [18,19,20,21].

**Figure 2:**
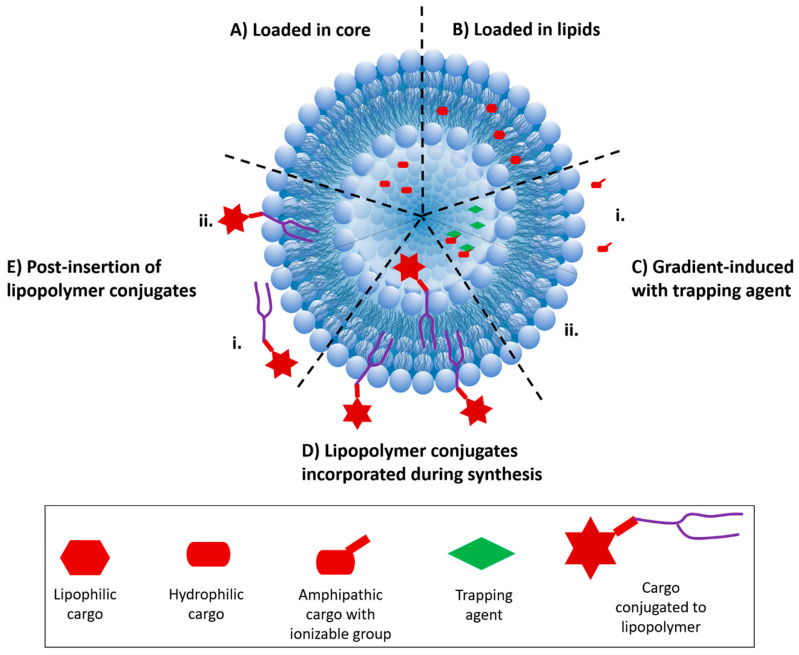
Various modes of liposomal drug incorporation. In passive loading, drugs can be incorporated into the nanoparticle (NP) during liposome synthesis. Depending on the drug’s properties, it can be loaded into (**A**) the hydrophilic core or into (**B**) the hydrophobic lipid bilayer. (**C**) In active loading, (i) drugs can be incorporated into the core of the liposome after NP synthesis by using pH or salt gradient, and (ii) after gradient-induced internalization, the drug forms a complex with the trapping agent (ions/salt) and remains sequestered in the core. (**D**) Liposomes comprised of drug-bearing lipopolymer conjugates displaying drugs on the surface and in the core are formed by incorporating the lipopolymer during NP synthesis. (**E**) Liposomes formed by post-insertion of the lipopolymer conjugate display the drug cargo on the NP surface.

Conventionally, drugs are incorporated into liposomes by either passive or active methods [22,23,24,25]. In passive incorporation, the drug of interest is dissolved in the organic phase with the phospholipid mixture, followed by solvent evaporation and thin film formation, which results in encapsulation of the drug in the aqueous core (for hydrophilic drugs) or the in the phospholipid bilayer (for hydrophobic drugs) when the liposome forms through hydration (Figure 2A,B). While this method offers relatively limited control over the selective loading of drugs into the specific real estate of the liposomes, its facile nature and ease of implementation allow for the incorporation of various drug molecules without further chemical modification. In active loading, the drug is loaded into as-synthesized liposomes by the establishment of a transmembrane pH gradient (i.e., the pH of the buffer environment inside and outside of the liposomes is different). The pH of the buffer outside the liposome allows the drug to exist in a stable amphipathic to enable its migration across the lipid bilayer into the aqueous core. This is followed by forming a complex between the drug and a ‘trapping’ agent present in the buffer inside of the liposome (Figure 2C) [24,25,26].

Active loading is efficient for many drugs (including doxorubicin) that possess an ionizable group or amphipathic properties [27]. Ionizable groups include ammonium sulfate, chloride ammonium, calcium acetate, sodium acetate, and polyanions such as polyphosphate, cyclodextrin, or sucrose octasulfate [28]. These groups can be used as “trapping” agents where their physicochemical properties allow them to bind to drug cargos and the solubility of the resulting complex can control the drug release rate from the liposomes [28]. This method of drug incorporation, however, cannot be applied to a large number of poorly water-soluble or insoluble drugs. In this instance, poor water solubility can be minimized by forming complexes between the drug and excipients such as cyclodextrin [29]. Another strategy to deal with poor solubility includes solvent-assisted active loading technology (SALT) [30]. Here, a small amount (5% *v*/*v*) of a water-miscible organic solvent (e.g., dimethyl sulfoxide) is used in the outside buffer to increase the solubility of the drug and assist the active loading, followed by the complete removal of the solvent by dialysis or gel filtration.

A third method for introducing the drug to liposomes is through chemical conjugation. Drugs with a proper chemical functional group or ‘handle’ can be attached to the phospholipid bilayer of the liposome by various chemistries. Drugs are covalently coupled to amphiphilic molecules such as PEGylated-lipids, polymers, or peptides that are able to interface with cognate functional groups on the phospholipid heads. Chemical conjugation is versatile and offers great control over both drug loading and the actuated release of the drug. Commonly used chemical linkages include disulfide, ester, and hydrazone that are labile and can release the drugs in the presence of reducing conditions, esterase enzymes, or low pH, respectively. Drugs can be conjugated to the liposome using two methods: 1—during the synthesis of the liposomes (preinsertion, Figure 2D) and 2—after liposome synthesis (post-insertion, Figure 2E). Incorporation by preinsertion results in drugs that are attached displayed both within the liposome core and on the surface of the liposomes, while post-insertion provides only outer surface attachment/display. Drugs can also be directly conjugated to the outer surface of the preformed liposomes. This strategy, however, requires careful consideration to ensure that the liposomal structure is compatible with the reaction conditions and post-conjugation purification protocols.

## 3. Strategies for Drug Incorporation and Liposome Bioconjugation/Targeting

In this section, we discuss examples from the recent literature wherein the various bioconjugation strategies discussed in Section 2 are used to incorporate drugs into and/or onto liposomes. In many of these examples, the bioconjugation strategies are designed to impart controlled drug release by any of a number of external stimuli. Various nanoparticles are often incorporated with the liposomes in order to achieve this responsiveness [31,32,33,34,35,36,37,38,39,40,41]. While some of the examples discussed here may include this stimuli-triggered response, our main focus in this review is a discussion of the bioconjugation strategies used to load/append drugs to the liposome. For readers interested in more detail on various methods for stimuli-triggered drug responses (including enzymatic, light, pH, temperature, and ultrasound), we direct the reader to the excellent reviews by Fouladi [42] and Parham [43].

### 3.1. Drug Incorporation during Liposome Synthesis

Incorporation of the drug cargo during liposome synthesis is the most commonly used strategy for introducing drugs to the liposome carrier. Here, we highlight two of the many examples in which drugs are incorporated during liposome synthesis. A more comprehensive list of examples is given in Table 1.

**Figure 3:**
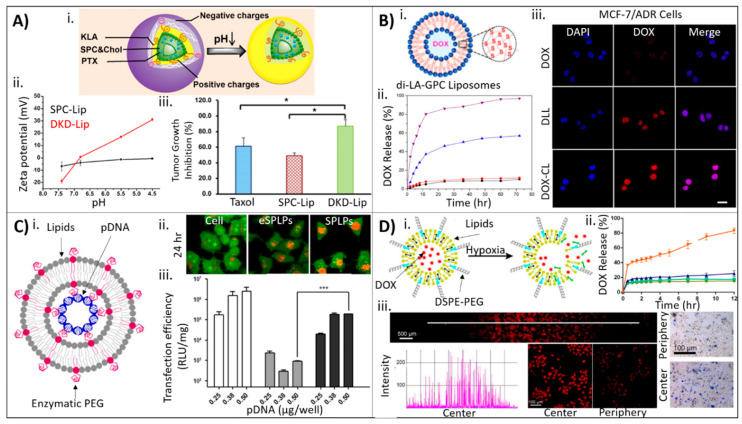
Drug incorporation during and after liposome synthesis. (**A**) (**i**) Illustration of DKD liposomes depicting change in surface charge from negative to positive with decreasing pH in the tumor microenvironment. (**ii**) Changes in zeta potential for non-reactive liposomes (black line) and DKD liposomes (red line) track with pH. (**iii**) Tumor growth inhibition for Taxol^®^ (blue), non-reactive liposomes (red), and DKD liposomes (green) shows inhibition of tumor growth for DKD liposomes (* *p* < 0.05). Image adapted from Jiang © Elsevier (2015) Ref. [44]. (**B**) (**i**) Schematic of di-LA-GPC cross-linked liposomes with DOX loaded into the core. (**ii**) DOX release for cross-linked liposomes (+GSH, purple), cross-linked liposomes (-GSH, red), non-cross-linked liposomes (blue), and Doxil^®^-like liposomes (black) shows only cross-linked liposomes (+GSH) and non-cross-linked liposomes release DOX. (**iii**) DOX-resistant MCF-7 cells treated with free DOX (top), Doxil^®^-like liposomes (middle), and cross-linked liposomes (bottom) show the greatest DOX release from cross-linked liposomes. Image adapted from Ling © Elsevier (2019) Ref. [46]. (**C**) (**i**) Schematic of enzyme-responsive liposomes loaded with plasmid DNA. (**ii**) Acridine orange assay for endolysomal disruption at 24 h for untreated cells only, enzymatically-responsive liposomes, and non-responsive liposomes showing decreased endosomal staining (orange) in responsive liposomes compared to non-responsive liposomes suggesting endosomal escape. (**iii**) Transfection efficiency of lipofectamine (white bars), non-responsive liposomes (gray bars), and enzyme-responsive liposomes (black bars) shows increased transfection efficiency (*** *p* < 0.001) for responsive liposomes. Image is from Song © Elsevier (2016) Ref.[49]. (**D**) (**i**) Schematic illustration of liposomes loaded with DOX showing disruption of liposomes in the presence of hypoxic cells via reduction of the nitroimidazole derivative. (**ii**) Cumulative DOX release over time for responsive liposomes in hypoxia (orange), responsive liposomes in normoxia (blue), non-responsive liposomes in hypoxia (green), and non-responsive liposomes in normoxia (yellow). (**iii**) FaDu cells cultured in conditions of normoxia at the periphery of the dish and hypoxia in the center show increased DOX release (red, left) and increased cell death (Trypan blue, right) in the center. Scale bare, 500 um (left) and 100 um (right). Image from Li © Elsevier (2019) Ref. [54].

Jiang and colleagues developed a multifunctional, mitochondria-targeted, pH-responsive liposome formulation of paclitaxel (PTX) [44]. To fabricate the liposomes, a proapoptotic peptide [KLAKLAK]_2_ (KLA) was modified with 2,3-dimethylmaleic anhydride (DMA) on the lysine (K) side chains via an amide bond. The modified peptide was subsequently covalently bound to the phospholipid 1,2-distearoyl-*sn*-glycero-2-phosphoethanolamine (DSPE), resulting in a DSPE-KLA-DMA (DKD) peptide–lipid hybrid. This custom lipid was mixed with soybean phosphatidylcholine (SPC), cholesterol, and PTX to form liposomes (150 nm in diameter) using the thin film hydration method (Figure 3A). DMA can impart charge-conversion to nanoparticle surfaces, and conjugation within the custom lipid did not affect this property; at low pH (pH 4.5–6.8) a surface charge conversion from negative to positive occurred that was attributed to the cleavable amide linkage formed between the lysine amine and DMA wherein the carboxyl groups transformed simultaneously into amino groups, a phenomenon the researchers state has previously been demonstrated in the literature. The researchers hypothesized that this increase in positive liposomal surface charge would facilitate cellular uptake and endosomal escape of the liposomes, resulting in homing to mitochondria and the induction of apoptosis. To confirm this hypothesis, the effects of the DKD liposomes were compared to liposomes without the DKD peptide. The results showed that cellular uptake of DKD liposomes increased 8-fold for human adenocarcinoma alveolar basal epithelial cells (A549) at pH 6.8 compared with pH 7.4 (neutral). The DKD liposomes were also significantly uptaken in Taxol^®^-resistant A549 cells; Taxol^®^ is an FDA-approved, clinical liposome containing PTX. Mitochondrial targeting was 4-fold greater for DKD liposomes compared to non-DKD liposomes in A549 cells, and increased to an 8.5-fold difference for Taxol^®^-resistant cells. Furthermore, in vivo, DKD liposomes were able to significantly inhibit tumor growth compared to both Taxol^®^ and non-DKD liposomes (87% vs. 62% and 49%, respectively; Figure 3A).

Utilization of the strong reducing conditions of the cellular cytosol, where glutathione (GSH) concentrations are 10 mM [45], has become a very effective way to mediate the release of drugs through the intracellular reduction of disulfide linkages. Ling and colleagues designed cross-linked liposomes capable of drug delivery under reducing conditions [46]. Here, the liposomes were fabricated by first conjugating lipoic acid (LA) and glycerophosphorylcholine (GPC) to form a dimeric LA-PC conjugate (di-LA-PC). This lipid was mixed with doxorubicin (DOX) and formed liposomes following thin film hydration, encapsulating DOX in the liposome core. During liposome fabrication, dithiothreitol (DTT) was added in order to cross-link the lipid bilayer using disulfide linkages (Figure 3B); the researchers hypothesized that GSH would result in reducing conditions that would mediate the degradation of the liposomes and release of the encapsulated DOX. The cross-linked di-LA-PC-DOX liposomes (170 nm diameter) were compared to non-cross-linked liposomes (150 nm) and Doxil^®^-like liposomes (265 nm). DOX encapsulation efficiency was 25% greater compared to non-cross-linked liposomes and was similar to Doxil^®^-like liposomes. The addition of GSH confirmed the responsiveness of the di-LA-PC-DOX liposomes to reducing conditions, demonstrating that cross-linked liposomes released DOX only in the presence of GSH to a significantly greater degree than the control liposomes. It was further demonstrated that the cross-linked liposomes preserved the efficacy of DOX, resulting in similar cytotoxicity in human breast adenocarcinoma cells (MCF-7) compared to control liposomes. In DOX-resistant cells (MCF-7/ADR), the cross-linked liposomes demonstrated increased uptake compared to control liposomes, resulting in a decrease in cellular viability compared to free DOX.

### 3.2. Drug Incorporation after Liposome Synthesis

The physicochemical properties of the drug cargo (e.g., charge, hydrophobicity, and size) often dictates at what point during the liposome synthesis process the drug can be introduced to the liposomal carrier [47,48,49,50]. In a recent study, Song and colleagues developed stabilized plasmid–lipid particles (SPLPs) composed of 1,2-dioleoyl-3-trimethylammonium-propane (DOTAP), 1,2-dioleoyl-*sn*-glycero-3-phosphoethanolamine (DOPE), and mPEG-GLFG-K-(C_16_)_2_, a polyethylene glycol (PEG)-modified lipid with an enzymatically degradable peptide linker [49]. The as-synthesized liposomes were incubated with plasmid DNA to promote passive loading into the liposome core. Following incubation, the liposomes were dialyzed against water then further separated using ion-exchange chromatography. Encapsulation efficiency was quantified using PicoGreen after dissociation of the SPLPs using Triton X-100 (Figure 3C). The liposomes exhibited diameters of 200 nm, loading efficiencies above 80% for the plasmid DNA, and high cellular viability compared with polyethylenimine. As SPLPs are often very stable, the researchers included a cathepsin B-cleavable PEG to control liposome degradation inside endosomes. Following endocytosis, the PEG shell was designed to be degraded by cathepsin B to expose DOPE to the endosome. As DOPE is fusogenic, this would result in enhanced degradation of the liposome and pDNA escape. Using an acridine orange assay for endo/lysomal disruption, the researchers demonstrated that the enzymatic liposomes had significantly decreased endosomal fluorescence compared to control liposomes, supporting their mechanistic hypothesis (Figure 3C). Further, transfection of human embryonic kidney cells (HEK293) showed increased pDNA transfection of the enzymatic SPLPs compared to non-enzymatic SPLPs, which the researchers attributed to the enhanced endosomal disruption and pDNA escape from enzymatic liposomes; the enzymatic liposomes (0.38–0.5 µg/well pDNA) transfected the HEK293 cells to similar levels as lipofectamine 2000 with 0.25 µg/well pDNA.

For drugs that do not interact well with the polar phosphate heads of liposomes, such as DOX, diffusion of the drugs within the liposome is mediated using gradients (e.g., salt [51] or pH [52,53]). Li and colleagues developed liposomes incorporating a nitroimidazole derivative for hypoxia-triggered drug delivery of DOX utilized pH gradients to facilitate the loading of the DOX [52]. The liposomes were composed of 1,2-dipalmitoyl-*sn*-glycero-3-phosphocholine (DPPC), cholesterol, 1,2-dimyristoyl-*sn*-glycero-3-phosphorylcholine (DMPC), 1,2-distearoyl-*sn*-glycero-2-phosphoethanolamine-*N*-[methoxy (polyethylene glycol)-2000] (DSPE-PEG2000), and a hypoxia-triggered nitroimidazole derivative. These liposomes ranged in diameter from 160 to 250 nm depending on the amount of incorporated nitroimidazole derivative, with DOX encapsulation efficiencies above 90%. In acute hypoxic conditions, the nitroimidazole derivative becomes reduced, causing the degradation of the liposome and release of the encapsulated DOX (Figure 3D). Indeed, when incubated in solutions containing Na_2_S_2_-O_4_, the liposomes fully degraded. To mimic hypoxia, the liposomes were incubated in solutions containing rat liver microsomes and NADPH. In these conditions, DOX release increased above a leakage threshold of 20% for the nitroimidazole derivative-containing liposomes. To demonstrate these effects in cells, the researchers designed an oxygen gradient system in which murine prostate cancer cells (RM-1) or human pharynx squamous carcinoma cells (FaDu) were cultured in dishes with a coverglass placed on top. The cells on the periphery of the dish (uncovered by the coverglass) experienced normoxia, whereas the cells in the center experienced hypoxia. Cells in the hypoxic (center) regions demonstrated a marked increase in red fluorescence compared to the normoxic periphery, which corresponded with DOX release. Further, there was an increase in apoptotic cells in the hypoxic (center) region (Figure 3D). In vivo, the hypoxia-responsive liposomes exhibited enhanced antitumor efficacy compared to non-responsive liposomes.

A different study by Plourde and colleagues used aptamers encapsulated within the liposomes to mediate DOX encapsulation within the liposomes. Here, DOX was still incorporated after liposome synthesis but this incorporation was achieved without the need for the traditionally used pH gradient [47]. Specifically designed DNA aptamers for DOX were incorporated within cationic liposomes composed of DOTAP, cholesterol, and DSPE-PEG2000. A non-cationic liposome control was formulated using 1-palmitoyl-2-oleoyl-*sn*-glycero-3-phosphocholine (POPC), cholesterol, and DSPE-PEG2000. Further, to compare against the current gold standard and liposomal formulations that incorporate DOX via pH gradients, the researchers also developed Doxil^®^-like liposomes fabricated using 1,2-distearoyl-*sn*-glycero-3-phosphocholine (DSPC), cholesterol, and DSPE-PEG2000. The researchers developed a range of DOX aptamers that varied in binding sites and nucleotides. By optimizing the ratio of aptamers to lipids, they achieved liposomes with greater than 90% aptamer encapsulation efficiency; subsequent DOX encapsulation efficiencies ranged from 28 to 86% depending on the aptamer used. The aptamer–liposomal formulations with the highest DOX encapsulation efficiencies were Doxapt-30 (84% DOX encapsulation) and Poly-Doxapt (86% DOX encapsulation), which had the closest DOX encapsulation efficiencies to Doxil^®^-like liposomes (98% DOX encapsulation). For blank liposomes (not Doxil^®^-like and no aptamers), DOX encapsulation efficiency did not increase beyond 5%. Further, by tuning the aptamer structure, the DOX release rate could be controlled, further demonstrating the potential of this method compared to other DOX encapsulation methods.

### 3.3. Drug Incorporation Using Covalent Conjugation

The covalent bioconjugation strategy is most often used in the formulation of prodrugs that are capable of self-assembly into liposomes [55,56,57,58,59,60]. Traditionally, the prodrug consists of a phospholipid that is covalently bound to a drug with a responsive element that mediates liposome degradation and drug release [61]. These liposomes may be further decorated with peptides or antibodies for increased targeting or drug responsiveness. Here, we will discuss two examples of lipid-drug prodrug liposomes designed to release the drug in specific conditions.

**Figure 4:**
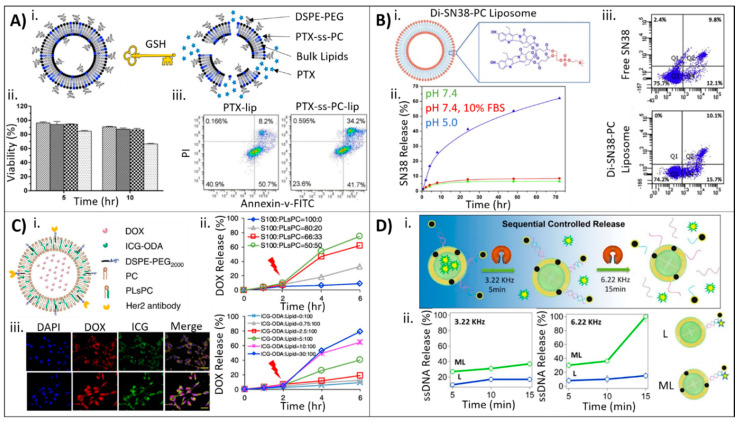
Examples of liposome–drug systems that incorporate drugs using covalent bioconjugation or a combination of bioconjugation methods. (**A**) (**i**) Schematic depicting liposomes composed of disulfide-containing lipid conjugate, PTX-ss-PC, demonstrating degradation and PTX release in the presence of GSH. (**ii**) Cellular viability of non-responsive liposomes without GSH (diagonal), responsive liposomes without GSH (small checkers), non-responsive liposomes with GSH (big checkers), and responsive liposomes with GSH (dots) demonstrating a decrease in MCF-7 viability over time for only responsive liposomes exposed to GSH. (**iii**) Flow cytometry analysis of annexin staining of MCF-7 cells (for apoptosis) after 48 h demonstrating increased apoptosis for responsive liposomes compared to non-responsive liposomes. Image adapted from Wang © Elsevier (2019) Ref. [56]. (**B**) (**i**) Schematic and scheme depicting liposomes fabricated with Di-SN38-PC prodrug. (**ii**) Cumulative SN38 release from Di-SN38-PC liposomes at pH 7.4 (green), pH 7.4 with 10% FBS (red), and pH 5.0 (blue) showing release only in acidic conditions. (**iii**) Flow cytometry analysis of apoptosis in MCF-7 cells at 36 h showing increased apoptosis for Di-SN38-PC liposomes compared with free SN38. Image from Du © Elsevier (2017) Ref. [58]. (**C**) (**i**) Schematic of responsive liposomes that degrade upon NIR excitation. (**ii**) DOX release over time as a function of liposome composition demonstrating that 33 mol % and 10 mol % of PLsPC lipid (top) and ICG-ODA (bottom) are required, respectively. (**iii**) MCF-7 cells treated with Her2-targeted responsive liposomes without laser (top) and with laser (bottom) stained for nuclei (blue), DOX (red), and ICG (green) demonstrating that NIR light increases DOX release. Scale bar, 20 um. Image adapter from Li © Elsevier (2018) Ref. [52]. (**D**) (**i**) Schematic illustration of magnetoliposomes showing sequential controlled release at different alternating magnetic fields. (**ii**) Release of ssDNA over time for magnetoliposomes (green) and blank liposomes (blue) at 3.33 kHz (left) and 6.22 kHz (right) showing that only magnetoliposomes, which contain iron oxide nanoparticles in the lipid bilayer, at 6.22 kHz release ssDNA. Image from Salvatore © American Chemical Society (2016) Ref. [66].

Wang and colleagues developed PTX-ss-lysophosphatidylcholine prodrugs (PTX-ss-PC) that degrade under reducing conditions [56]. The lipid-drug conjugates were synthesized following a five-step esterification process, and were mixed with DSPE-PEG2000, cholesterol, and 1,2-dioleoyl-*sn*-glycero-3-ehtylphosphocholine (EPC) to form liposomes approximately 210–235 nm in size. Incorporation of triphosgene in the prodrug imparted the sensitivity to reducing conditions, and GSH was used to trigger liposomal degradation (Figure 4A). Control liposomes consisted of the same lipid formulation without the PTX prodrug (PTX loaded following traditional protocols). Only the responsive prodrug liposomes degraded and released PTX in solutions containing GSH. Further, prodrug liposomes showed increased uptake compared to control liposomes in MCF-7 cells, and this uptake was accompanied by decreased MCF-7 viability only when GSH was added, which indicates the triggered response of the prodrug liposomes. Viability of cells treated with the prodrug liposomes was comparable to Taxol^®^ after 48 h (79.3% apoptosis for Taxol^®^ compared to 75.9% for the prodrug liposome). Notably, Taxol^®^ is not a triggered release system, highlighting the superiority of this bioconjugation method.

In a different study, Du and colleagues developed a prodrug from 7-ethyl-10-hydroxycamptothecin (SN38), which is a highly active topoisomerase I inhibitor, and l-α-glycerophosphorylcholine (GPC) [58]. The prodrug was designed to contain two SN38 molecules per lipid (Di-SN38-PC) and was conjugated following a two-step process (Figure 4B). Using thin film hydration, the prodrug formed pH-responsive liposomes approximately 140 nm in size that degraded and released SN38 only in the presence of an acidic environment (pH 5.0). Prodrug synthesis and liposomal fabrication significantly improved MCF-7 uptake of SN38 at earlier time points (3X greater uptake at 3 h compared with free SN38). Further, conjugation to the lipid and self-assembly into a liposome did not negatively affect the cytotoxicity effects of SN38, as the results clearly demonstrate similar cell viability in MCF-7 cells for Di-SN38-PC liposomes compared with free SN38 (apoptosis percentages of 16.2% and 21.9% for free SN38 at 24 and 36 h, respectively, and 10% and 25.8% for Di-SN38-PC liposomes at 24 and 36 h, respectively). Finally, the liposomal SN38 formulation demonstrated increased blood retention time, increased maximum SN38 concentrations, and a longer half-life compared with free SN38 in albino BALB/c mice.

### 3.4. Drug Incorporation Using a Combination of Strategies

Many liposomal formulations utilize a combination of bioconjugation strategies for drug loading and control of drug release/activity in order to achieve complex liposomal designs capable of incorporating multiple drugs or achieving multiple goals (e.g., trigger drug release, modulate drug activity, and/or target specific cells of interest) [52,62,63,64,65,66,67]. The most common combinations are incorporation during synthesis and incorporation by passive diffusion after liposome synthesis and incorporation during synthesis and covalent bioconjugation strategies.

By incorporating drugs both during liposome synthesis and passively after synthesis, researchers can load liposomes with drugs of significantly different physicochemical properties to realize combinatorial therapies. For example, in a recent study by Deng and colleagues, X-ray triggered liposomes were designed that incorporated both gold nanoparticles (AuNPs) and the photosensitizer verteporfin (VP) for a combinatorial approach combining radiotherapy with chemotherapy [64]. The liposomes were fabricated by mixing DOTAP, 1,2-dioleoyl-*sn*-glycero-3-phosphocholine (DOPC), AuNPs, and VP following a thin film hydration method, and DOX was incorporated after liposome fabrication using a pH gradient exchange method, as previously discussed. The liposomes loaded with both AuNPs and VP demonstrated an enhanced response to X-ray and singlet oxygen generation, resulting in increased calcein release, which was used as a model drug. In a xenograft colorectal cancer mouse model, no tumor growth was seen for tumors treated with X-ray triggered liposomes encapsulating DOX, whereas non X-ray triggered liposomes containing DOX or X-ray alone resulted in a 3–4× increase in tumor growth. The X-ray triggered liposomes were also capable of gene silencing when encapsulated with antisense oligonucleotides.

Li and colleagues developed a photosensitive liposome capable of releasing DOX in the presence of NIR light for combined photodynamic-chemotherapy [52]. Two types of liposomes were fabricated. The first set of photosensitive liposomes were composed of indocyanine green-octadecylamine (ICG-ODA), soybean lecithin (S100), 1-(1z-octa-decenyl)-2-oleoyl-*sn*-glycero-3-phosphocholine (PLsPC), cholesterol, and DSPE-PEG2000 and fabricated using the thin film hydration method. DOX was incorporated passively after liposome synthesis using a pH gradient. The second type of liposome contained a HER2-targeting antibody in addition to the other components. In these liposomes, DSPE-PEG200-NH-DSC was mixed with ICG-ODA, S100, PLsPC, cholesterol, and DSPE-PEG2000 to form liposomes, after which the DSC was coupled with the HER2 antibodies to form targeted photosensitive liposomes (Figure 4C). In both liposomal formulations, the photosensitive moieties that resulted in the degradation of the liposome and formation of the singlet oxygen for photodynamic therapy were the PLsPC lipid and the hydrophobically-modified photosensitizer ICG-ODA, which further acted as an optical imaging tool. The researchers achieved high DOX encapsulation efficiencies (>80%) even when varying lipid molar ratios. Beyond a small amount of DOX leakage, DOX release was demonstrated primarily in only photosensitive liposomes containing at least 33 mol % PLsPC and 10 mol % ICG-ODA upon excitation with NIR light (Figure 4C). Further, release of DOX within in vivo tumors or in vitro in MCF-7 cells was only demonstrated for photosensitive liposomes after NIR excitation (Figure 4C). Finally, ROS generation was also demonstrated to be directly related to the composition of the liposomes, with greater ROS generation in liposomes with higher amounts of ICG-ODA.

When combining covalent bioconjugation strategies with incorporation of drugs during synthesis, complex liposomal formulations may be achieved. Salvatore and colleagues developed multifunctional magnetoliposomes that were capable of releasing cargo sequentially as a result of the magnetic field power used [66]. In this study, iron oxide nanoparticles were first synthesized then mixed with Au, 1,2-hexadecanediol, oleic acid, and oleylamine to form core–shell nanoparticles. These nanoparticles were then mixed with ssDNA and chol-DNA to form a core–shell nanoparticle–DNA dispersion. Finally, this was mixed with liposomes either containing iron oxide nanoparticles encapsulated in the lipid membrane or blank liposomes to form magnetoliposomes (Figure 4D). These liposomes could also be encapsulated further with hydrophilic drugs in the core. Stimulation with an alternating magnetic field (AMF) at 3.22 kHz resulted in the destabilization of the lipid membrane and release of the encapsulated hydrophilic drug. Further stimulation at 6.22 kHz resulted in the degradation and release of the DNA layer. The degradation and release of the drug/DNA from the liposomes could be visualized using fluorescence correlation spectroscopy (FCS). FCS confirmed that stimulation at 3.22 kHz was not sufficient to release ssDNA, but 6.22 kHz was. Further, FCS confirmed that the iron oxide nanoparticles encapsulated within the lipid bilayer aided in increasing ssDNA release compared with blank liposomes. Complimentary studies looking at carboxyfluorescein (CF) release, used as a model hydrophilic drug, further demonstrated release of CF at 3.22 kHz but not 6.22 kHz and only when iron oxide nanoparticles were embedded within the lipid bilayer.

**Table 1:** Examples of liposomes in literature from 2018 to 2020 using bioconjugation strategies for drug encapsulation and targeting.

Drug Loading Method	Drug	Targeting Moiety	Liposome Composition	Application	Ref.
Incorporation during synthesis	Porphyrins (p-NH2, pOH, p-py)	HA (polymer)	DPPC, DOPG, chol-hy, HA	Delivery of photosensitizer porphyrins to CD44+ cells	[68]
	ND	FA (vitamin)	FA-PEG-DSPE, ND and DSPC, chol, mPEG-DSPE	Treatment of P-glycoprotein+ and FA receptor+ tumors	[69]
	Anidulafungin	α-tocopherol (vitamin)	HSPC, PG, chol	Treatment of fungal infection	[70]
	DOX	FA (vitamin)	SPC, chol, DSPE-PEG or FA-PEG-DSPE	Comparison of two liposome fabrication methods for DOX	[71]
	Goniodiol	N/A	DSPC, PEG-A-DSPE or PEG-P-DSPE	Improved stability and activity of goniodiol for cancer treatment	[72]
	7,8-DHF	N/A	SPC, chol, LF	Effects of crosslinking on drug release	[73]
	Calcein, TPS	N/A	DSPC, DOPE, chol, DSPE-PEG2000	Light-triggered drug release for cancer	[74]
	17β-estradiol	N/A	DPPC, DMPC or POPC, DDAB, chol, DSPE-PEG2000	Preventing activation of undesired pathways while retaining drug activity	[75]
	Essential oils (e.g., estragole, isoeugenol, eucalyptol, pulegone, terpineol, thymol)	N/A	Lipoid S100, chol	Improved shelf life and activity	[76]
	KSP siRNA, PTX	N/A	DC-chol, DOPE, mPEG2000-DSPE	Dual-drug delivery for multi-drug resistant ovarian cancer tumors	[77]
	DOX	N/A	HSPC, corosolic acid, DSPE-PEG2000	Increased cancer cell drug uptake and treatment	[78]
	DOX	N/A	di-LA-GPC prodrug	Improved liposome stability for cancer	[46]
	PTX-BSA	N/A	HEPC, DSPE-PEG2000	Improved drug encapsulation and antitumor effect	[79]
	Curcumin	N/A	Soybean lecithin, chol, chitosan	Increased liposome stability	[80]
	Gemcitabine-copper(II) gluconate complex	N/A	DPPC, DSPC, DSPE-PEG2000	Heat-triggered drug delivery	[81]
	RFP, CaO_2_	N/A	DSPE-PEG3400, lecithin, lactic acid, stearic acid, PCM	Bacteria toxin-triggered antibiotic release	[82]
	Iridium(III) polypyridyl complexes	N/A	PC-98T:CHO-HP, PC-98T:DSPE-mPEG2000	Improving anticancer efficacy of iridium(III) polypyridyl complexes	[83]
Incorporation after synthesis	DOX	Porphyrins	DSPC, chol, DSPE-PEG, DOPC	Ultrasound-triggered, localized DOX release	[84]
	Usnic Acid	N/A	Cationic or N-oxide surfactants, DMPC, chol	Improved antioxidant delivery	[48]
	Ciprofloxacin	N/A	DSPC, chol, DOPC, porphyrin-phospholipid, mPEG-2000-DSPE	Light-triggered antibiotic treatment	[50]
	DOX	N/A	DSPE-PEG2000, nitroimidazole, DPPC, chol, DMPC	Hypoxia-triggered DOX release	[54]
	DOX and Irinotecan	N/A	DSPC, chol, mPEG2000-DSPE	Combination treatment for treating cancer	[51]
	Sinomenine hydrochloride	N/A	DPPC, SPC, chol	Heat-triggered drug release for rheumatoid arthritis	[53]
Covalent attachment to liposome surface	APL9 peptide	F4/80 (antibody)	PAM_3_CysSK_4_-peptide	Modified immune response in type 1 diabetes	[59]
	GALA peptide	tbFGF lipopeptide	POPC, DPTE-lipopeptides	Endosomal escape and cell targeting	[57]
	Camptothecin	N/A	Di-CPT-GPC prodrug	CPT prodrug for anticancer treatment	[55]
	PTX	N/A	PTX-ss-PC prodrug, mPEG2000-DSPE, EPC, chol	Reduction-triggered, intracellular delivery	[56]
	Artesunate	N/A	Di-ART-GPC	Anti-inflammatory treatment of rheumatoid arthritis	[60]
Combination of drug loading strategies	DOX	HER2 (antibody)	ICG-ODA, DSPE-PEG2000, PLsPC, S100	Light-triggered drug release and ROS generation for chemotherapy	[52]
	Calcein	FA (vitamin)	DOTAP, DOPC, AuNPs, VP	X-ray-triggered drug release for radiotherapy and chemotherapy	[64]
	DOX, Bcl-2 siRNA	N/A	TPGS or PEG-DSPE, DOTAP, DPPC, chol	Chemotherapy with dual suppression of drug resistance	[62]
	Gd-DTPA, DOX	N/A	Gd-DTPA-ONB	MRI-guided liposome drug delivery	[67]
	Disulfiram and DOX	N/A	DSPC, chol, mPEG2000-DSPE	Inhibit/reverse multidrug resistance in cancer cells	[63]
N/A	N/A	HER2 (antibody)	DSPE-PEG2000, DPPC, chol	Improved targeting of HER2 cancer cells	[85]
	N/A	HER2 (antibody)	FcBP, PEG-DSPE	Antibodies to increase targeting affinity	[86]
	N/A	CD11c (antibody)	DOPE, EPC, chol, DBCO-PEG	SPAAC modification	[87]

Abbreviations: 7,8-DHF, 7,8-dihydroxyflavone; APL9, an altered version of the GAD546−554 (glutamic acid decarboxylase 65) peptide; AuNPs, gold nanoparticles; Bcl-2, cellular antiapoptotic protein; BSA, bovine serum albumin; CA, corosolic acid; CD11c, integrin alpha X; CD44, a cell-surface glycoprotein used as a cancer marker; CHO-HP, cholesterol; chol, cholesterol; chol-hy, hydrazide-cholesterol; DBCO-PEG, Dibenzylcyclooctyne polyethylene glycol; DC-cholesterol, 3β-[*N*-(*N*′,*N*′-dimethylaminoethane) carbamoyl] cholesterol; di-ART-GPC, dimeric artesunate phospholipid conjugate; di-CPT-GPC, dimeric camptothecin glycerophosphorylcholine; di-LA-GPC, dimeric lipoic acid-glycerophosphorylcholine; DDAB, Dimethyldioctadecylammonium Bromide Salt; DMPC, 1,2-dimyristoyl-d54-*sn*-glycero-3-phosphocholine; DOPC, dioleoylphosphatidylcholine; DOPE, 2-dioleoyl-*sn*-glycero-3-phosphoethanolamine; DOPG, 1,2-dioleoyl-*sn*-glycero-3[phospho-rac-(1-glycrol)]; DOTAP, 1, 2-di-(9*Z*-octadecenoyl)-3-trimethylammonium-propane; DOX, doxorubicin; DPTE, 1,2-dipalmitoyl-*sn*-glycero3-phospho-thio-ethanol; DPPC, di-palmitoylphosphatidylcholine; DSPC, 1,2-distearoyl-*sn*-glycero-3-phosphocholine; DSPE, di-stearoylphosphatidylethanolamine; DSPE-PEG2000/DSPE-PEG, PEG-2000 di-stearoylphosphatidylethanolamine; DSPE-PEG3400, PEG-3400 di-stearoylphosphatidylethanolamine; EPC, L-α-phosphatidylcholine; FA, folate/folic acid; FA-PEG-DSPE, Folate-polyethylene glycoldistearoylphosphatidylethanolamine; FcBP, Fc binding peptide; F4/80, F4/80 macrophage protein; GALA, a pH sensitive fusogenic peptide; Gd-DTPA, an MRI contrast agent; HA, hyaluronic acid; HEPC, hydrogenated egg yolk phosphatidylcholine; HER2, human epidermal growth factor receptor 2; HSPC, hydrogenated soy phosphatidylcholine; ICG-ODA, a hydrophobically modified photosensitizer composed of indocyanine green (ICG) and octadecylamine (ODA); KSP, kinesin spindle protein; LF, lactoferrin; mPEG(2000)-DSPE, 1,2-distearoyl-*sn*-glycero-3-phosphoethanolamine-*N*-[amino(polyethylene glycol)-2000]; N/A, no targeting moiety/drug used; ND, nitrooxy-Dox derivative; ONB, o-nitro-benzyl ester; PAM_3_CysSK_4_, *N*-Palmitoyl-*S*-[2,3-bis(palmitoyloxy)-(2*R*,*S*)-propyl]-(*R*)-cysteinyl-seryl-(lysyl)3-lysine; PCM, phase change material; PC-98T, egg yolk lecithin; PEG, polyethylene glycol; PEG-DSPE, 1,2-distearoyl-*sn*–glycero-3-phosphoethanolamine-*N*-[methoxy (polyethylene glycol)−2000]; PEG-A-DSPE, acetamide-linked PEG-DSPE conjugate; PEG-P-DSPE, pentanamide-linked PEG-DSPE conjugate; PG, phosphatidylglycerol; PLsPC, 1-(1z-octadecenyl)-2-oleoyl-*sn*-glycero-3-phosphocholine; POPC, 1-Palmitoyl-2-oleoyl-*sn*-glycero-3-phosphocholine; p-NH2, 5,10,15,20-tetrakis(4-aminophenyl) porphyrin; p-OH, 5,10,15,20-tetrakis(4-hydroxyphenyl) porphyrin; p-py, 5,10,15,20-tetra(4-pyridyl) porphyrin; PTX, paclitaxel; PTX-ss-PC, disulfide derivative paclitaxel-ss-lysophosphatidylcholine prodrug; RFP, rifampicin; ROS, reactive oxygen species; SPAAC, strain-promoted alkyne–azide cycloaddition; SPC, soybean lecithin; S100, soya bean lecithin; tbFGF, truncated basic Fibroblast Growth Factor; TPGS, d-α-Tocopherol polyethylene glycol 1000 succinate; TPS, talaporfin sodium; VHH, a single variable domain on HER2 antibody; VP, verteporfin.

## 4. Bioconjugation Strategies for Targeting and Delivery of Liposomes to Cells

Just as the loading of the liposomal carrier with the drug cargo is critically important for enhanced drug efficacy, the delivery and targeting of the liposome to cells is equally important. Initial strategies relied on the enhanced permeability and retention (EPR) effect wherein NPs of certain sizes tend to accumulate within the leaky vasculature that is common to many tumor types [88]. Current literature reports have sought to improve specificity of liposome targeting by utilizing bioconjugation methods in which liposomes are conjugated to various ligands (peptides, proteins, and small molecules) at the liposome surface. These ligands impart distinct properties (e.g., charge, receptor specificity and membrane insertion) that enhances the liposome’s ability to deliver its drug payload to the intended therapeutic target [89,90]. Bioconjugation of ligands to liposomes increases cell-type specificity, decreases off-target effects, and often enables the administration of lower (and fewer) dosages of dug. In this section, we discussed select examples from the recent literature that highlight various bioconjugation strategies for the cellular targeting of liposome–drug composites and more examples are listed in Table 1.

### 4.1. Antibody–Liposome Bioconjugates (Immunoliposomes)

The surface attachment of antibodies to liposomes is a common approach used to produce actively targeted drug delivery systems. This liposomal formulation allows for efficient targeting of the antibody–liposome bioconjugate to its matching antigen. Once the immunoliposome has docked at the site of the antigen, it can deliver its drug payload, thus minimizing off-target effects of the drug. Bioconjugation methods for attaching antibodies to liposomal surfaces typically involve covalent bonding of the antibody to the liposome surface [91]. Much of the current literature focuses on examining new antibody designs or antibody–drug variations for therapeutic applications [92,93,94,95]. For example, Nikkhoi and colleagues explored the development of a bivalent, bispecific VHH-domain HER2-antibody fragment attached to liposomes to treat HER2-positive breast cancer [85]. The VHH antibody fragments were attached to the surface of PEGylated liposomes using a thiol-maleimide reaction activated with N-hydroxysuccinimide (NHS), which is a frequent strategy of attachment for antibodies. The group was then able to demonstrate that their bivalent, bispecific antibody–liposomes exhibited the greatest affinity towards HER2-positive cells as compared to monovalent and bivalent, monospecific VHH antibody–liposome conjugates due to their ability to target the different HER2 epitopes.

**Figure 5:**
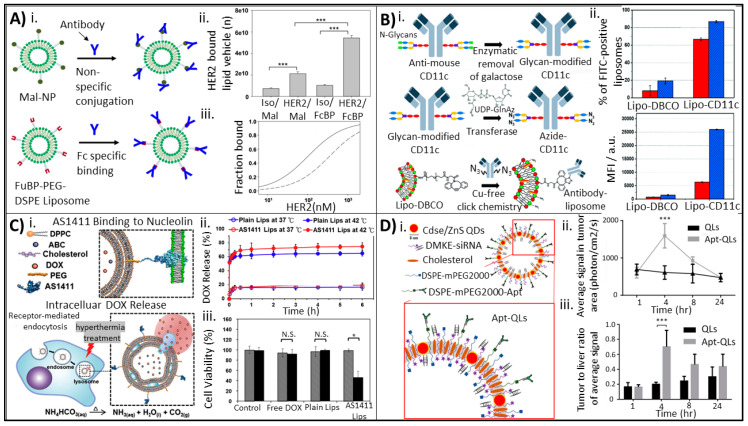
Bioconjugation strategies using antibodies and aptamers for improved drug targeting and delivery. (**A**) (**i**) Schematic of antibody orientation on maleimide-PEG-DSPE NPs versus Fc-specific binding onto *Staphylococcus aureus* and FcBP-PEG-DSPE NPs. Antibodies bind nonspecifically to Mal-NPs while Fc-specific binding leads to oriented control in *S. aureus*. Conjugation of FcBP to liposome surface allows for controlled orientation of antibodies on engineered NPs. (**ii**) Quantification of HER2 bound to the fluorescent liposome. NPs with the HER2 antibody had significantly increased binding versus NPs with the control IsoIgG antibody (*** *p* < 0.05) and HER2/FcBP-NPs had significantly increased binding versus HER2/Mal-NPs. (**iii**) Microscale thermophoretic binding curves for HER2/Mal-NPs and HER2/FcBP-NPs versus concentration of the HER2 protein. Dotted line denotes HER2/Mal-NP, solid line denotes HER2/FcBP-NP. Image adapted from Shim © Elsevier (2019) Ref. [86]. (**B**) (**i**) Schematic of antibody functionalization and attachment via copper-free click chemistry to DBCO-surface modified liposomes. (**ii**) Confirmed antibody presence on the liposome surface. FITC-labeled secondary antibody was allowed to attach to DBCO and CD11c-conjugated liposomes under two different reaction conditions (blue versus red bars). Flow cytometry confirmed significantly higher presence of FITC labeling on CD11c-liposomes. Legend for both images: red denotes DBCO:NH_2_ = 1:1, blue denotes DBCO:NH_2_ = 3:1. Image adapted from Gai © Royal Society of Chemistry (2020) Ref. [87]. (**C**) (**i**) Schematic of the thermoresponsive AS1411 liposome with encapsulated doxorubicin and ABC, and its mechanism of nucleolin binding via AS1411 targeting and hyperthermia-induced intracellular doxorubicin release. (**ii**) Doxorubicin release of AS1411 liposomes suspended in aqueous media at body temperature (37 °C) or hyperthermic temperature (42 °C). (**iii**) MCR-7/ADR cell viability determined by the MTT assay when treated with control (untreated), free DOX, plain liposomes (containing DOX) or AS1411 liposomes (* *p* < 0.05). Grey depicts cells signified at 37 °C, black depicts cells signified at 37 °C; *n* = 6. Image adapted from Liao © Elsevier (2015) Ref. [104]. (**D**) (**i**) Schematic of EGFR-targeted, quantum dot and siRNA-carrying Apt-QL. (**ii**) Average fluorescent signal (generated from CdSe/ZnS Q-dots) in tumor xenograft within mice injected with QLs or Apt-QLs (*** *p* < 0.001). (**iii**) Tumor to liver ratio (average signal) in tumor xenografts in mice shows a significantly higher collection of Apt-QLs in tumor at 4 h after intravenous administration (*** *p* < 0.001). Image adapted from Kim © Nature Research (2017) Ref.[103].

Several studies have focused on the improvement of antibody–liposome conjugation strategies specifically to increase the antibody–antigen coupling efficiency. For example, a recent study by Shim and colleagues developed a new method of antibody–liposome conjugation mimicking the ability of *Staphylococcus aureus* bacteria in expressing surface-oriented proteins [86]. Currently, most research uses covalent methods for antibody–liposome attachment, but the researchers are then unable to control antibody orientation on the liposome surface. As a result, the liposome–antibody conjugate contains many misaligned antibodies and the antigen-binding fragment (Fab) is unable to bind to nearby antigens. To address this issue, Shim and colleagues decorated their liposomes with an FcBP (Fc binding peptide) found in *Staphylococcus aureus* that can differentiate between the Fab and Fc regions of the antibody. The FcBP was able to bind noncovalently to the Fc region of the antibody, allowing the Fab portion of the antibody to protrude externally. The researchers were able to develop FcBP-liposomes by first synthesizing an FcBP-conjugated lipid, which was done by amide bond formation. The FcBP lipids were formed into liposomes using thin film hydration. Finally, the FcBP-liposomes were incubated with the desired antibody (antiHER2) and purified by dialysis to produce orientation-controlled, antibody-tagged liposomes (Figure 5A). As a control group, HER2 antibodies were attached to maleimide-functionalized PEG-DSPE nanoparticles using a thiol-maleimide reaction, which is a common approach for antibody conjugation to liposomes. The researchers demonstrated that the HER2/FcBP-liposomes had a significantly higher HER2-binding affinity than HER2/Mal-liposomes formed via thiol-maleimide reaction by measuring the binding of fluorescent liposomes to HER2 coated plates (Figure 5A). Additionally, the researchers examined the cellular uptake of the liposomes in HER2-overexpressing BT-474 human ductal carcinoma cells. They used fluorescence microscopy to show that cellular uptake of HER2/FcBP-liposomes was notably higher than cellular uptake of HER2/Mal-liposomes. These results were further examined with a microscale thermophoresis technique, which determined that HER2/FcBP-liposomes had a 3.6 fold higher affinity to the HER2 protein versus HER2/Mal-liposomes (Figure 5A). Taken together, these results suggest that use of antibody-orienting technologies when considering liposome conjugation may lead to improved cell targeting and potency.

Another recent study established a novel method to conjugate antibodies to preformed liposomes [87]. This strategy was explored to improve the conjugation of antibodies to preformed liposomes (such as natural carriers), and to provide an alternate strategy of conjugation in the cases where prefunctionalization disrupts the liposome self-assembly process. Their strategy involved the use of bioorthogonal copper-free click chemistry to couple antibodies to the surface of amino group-terminated liposomes. This research group developed DOPE, l-α-phosphatidylcholine (egg PC) and cholesterol liposomes using thin film hydration followed by extrusion. Next, dibenzylcyclooctyne-polyethylene glycol (DBCO-PEG) was conjugated to the liposome surface using an NHS ester conjugation reaction. CD11c-antibodies were prepared for conjugation via enzymatic removal of galactose from the Fc part of the antibody. Then, azide groups were attached to the antibody by incubation with UDP-N-azidoacetylgalactosamine (UDP-GalNAz; Figure 5B). Finally, azide-modified CD11c-antibodies were attached to the DBCO-liposomes using strain promoted alkyne-azide cycloaddition, which is a type of bioorthogonal copper-free click chemistry used in biological applications. Although the researchers did not complete affinity binding studies to determine the efficacy of CD11c-liposome targeting to cells, they were able to clearly show successful conjugation of the antibody to a preformed liposome surface using this novel method (Figure 5B).

### 4.2. Aptamer–Liposome Bioconjugates

Aptamers are single-stranded, short DNA or RNA oligonucleotides that can selectively bind to a single target. They are developed using an iterative enrichment technique known as systematic evolution of ligands by exponential enrichment (SELEX) that identifies optimal aptamer candidates from a larger oligonucleotide library. Aptamers have emerged as promising drug-targeting ligands due to their versatility, high affinity to a single target, small size, stability, low immunogenicity, and simple synthesis [90,96,97], which also makes these aptamers optimal candidates for liposome bioconjugation. Typically, aptamers are attached to liposomes using covalent bonding (preconjugation to lipids) or a post-insertion method. Covalent bonding techniques commonly employ 1-ethyl-3-(3-dimethylaminopropyl)carbodiimide (EDC) chemistry and thiol-maleimide linkage reactions [98,99,100,101]. The post-insertion method reacts functional group-activated lipids to aptamers to create unstable micelles, which are then mixed with preformed liposomes to incorporate the aptamer–lipids into the liposome membrane [97,102,103].

An example of covalent aptamer conjugation is demonstrated by Liao and colleagues, where they conjugated AS1411 antinucleolin aptamers to liposomes in order to target breast cancer cells [104]. The researchers conjugated thiolated AS1411 aptamers to maleimide-PEG 2000-DSPE using a thioether linkage. The conjugates were hydrated using aqueous ammonium bicarbonate (ABC) and remote-loaded with DOX post-formation of the liposomes. The research team hypothesized that this system would allow for thermoresponsive, tumor-specific chemotherapy where the AS1411 aptamer would allow cells to target breast cancer cells overexpressing nucleolin. Once at the tumor site, mild heating could be applied to the tumor to cause decomposition of ABC, causing CO_2_ bubble generation to disrupt the liposomal bilayer and locally release DOX (Figure 5C). The researchers were able to show the liposomes containing ABC were thermosensitive at 42 °C and released the encapsulated DOX (Figure 5C). They also showed that MCF-7/ADR breast cancer cells had significantly lower viability when treated with AS1411 liposomes at 42 °C compared to treatment with free DOX or plain liposomes without the AS1411 aptamer (Figure 5C). An in vivo study using MCF-7/ADR breast cancer cells subcutaneously implanted into mice showed that the AS1411 liposomes, after heating to 42 °C, were able to deliver significantly more DOX to the tumor site versus plain liposomes and free DOX.

Kim and colleagues utilized a post-insertion approach wherein they developed cancer-targeted liposomes by conjugating antiEGF-receptor (EGFR) aptamers carrying quantum dots (QDs) and therapeutic siRNA to the liposome surface for a theranostic strategy [103]. Liposomes were prepared using thin film hydration, incorporating CdSe/ZnS QDs and water-soluble Bcl-2 siRNA passively during synthesis. Subsequently, thiolated antiEGFR aptamers were conjugated to DSPE-PEG2000-maleimide via the thioether bond and then added to liposomes using the post-insertion method described previously (Figure 5D). The researchers used MDA-MB-231 EGR-receptor positive and MDA-MB-453 EGFR negative human breast metastatic carcinoma cells to determine the efficacy of their strategy in targeting EGFR positive cells. Using fluorescence microscopy, the researchers determined that EGFR-positive cells showed significantly higher localization of QDs compared to EGFR-negative cells, with EGFR-positive cells treated with aptamer–QD–liposomes showing the highest transfection of QDs and siRNA. In an in vivo cancer model of a mouse injected with MDA-MB231 cells to create cancerous xenografts, Kim and colleagues further showed that mice injected with aptamer–QD–liposomes showed significantly higher average fluorescent signal in the tumor at 4 h as opposed to the QD–liposomes without aptamers (Figure 5D).

### 4.3. Peptide–Liposome Bioconjugates

Aside from antibodies or aptamers, peptide motifs that convey targeting affinity may also be used, including Asn-Gly-Arg (NGR) peptides [105,106] or Arg-Gly-Asp (RGD) peptides [107,108]. Peptides can be conjugated to the liposome surface by way of various covalent linkages, including maleimide-thiol bonds, peptide bonds, sulfanyl bonds, disulfide bonds, and phosphatidyletanolamine-linker bonds [109]. Additionally, larger peptides, proteins, and enzymes can be attached to the liposome surface to provide a desired functionalization [110,111].

**Figure 6:**
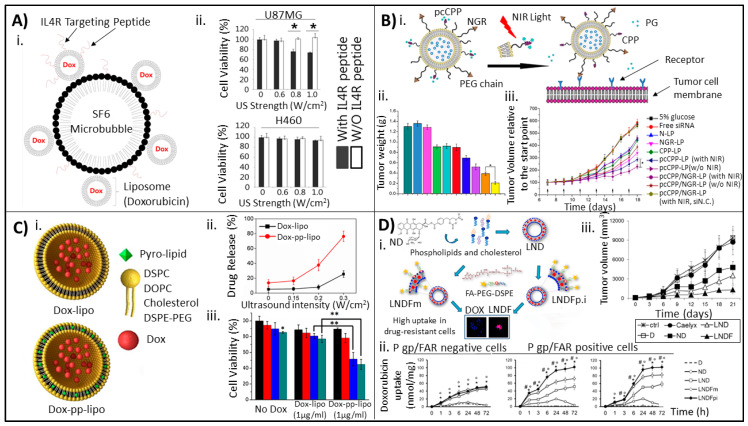
Liposomal bioconjugation strategies utilizing peptides and other small molecules for improved drug delivery and targeting. (**A**) (**i**) Schematic of MB-liposome (Dox) IL4RTP. (**ii**) Demonstrated inhibition of IL4R-expressing cell proliferation (U87MG) when treated with MB-lipo (Dox) at varying strengths of ultrasound with or without IL4R peptide attached. Comparison is shown to H460 (IL4R expression low) is shown, demonstrating the MB-lipo (Dox) IL4RTP primarily affects cells when ultrasound strength > 0.8 W/cm^2^ and cells are IL4R-expressing. Cell viability was determined via the WST-1 assay (* *p* < 0.001). Image adapted from Park © Spandidos Publications (2016) Ref. [112]. (**B**) (**i**) Schematic of depicting the siRNA-carrying pcCPP/NRG-LPs. The dual-modified liposomes are targeted to the tumor via the NGR ligand and upon activation with NIR light once at the tumor site, the photosensitive group (PG) is released, activating the CPP and allowing liposome entry into the cell. (**ii**) Weight of xenograft tumors after 10 days treatment (treatment began once tumors grew to approximately 50 mm^3^). Legend: teal; 5% glucose, blue, Free siRNA; magenta, pcCPP/NGR-LP (with NIR, siN.C.); green, N-LP; turquoise, pcCPP-LP (without NIR); red, CPP-LP; cobalt, pcCPP/NGR-LP (without NIR); purple, NGR-LP; orange, pcCPP-LP (with NIR); yellow, pcCPP/NGR-LP (with NIR). (* *p* < 0.05). (**iii**) Tumor volume relative to volume at the start point of treatment over days (* *p* < 0.05). (**iii**) Image from Yang © Elsevier (2015) Ref. [105]. (**C**) (**i**) Diagram of Dox-lipo versus Dox-pp-lipo (liposomes containing porphyrin within the membrane) for sonodynamic therapy. (**ii**) Dox release profile of Dox-lipo versus Dox-pp-lipo under increasing ultrasound intensities. Legend: black, no ultrasound; red, 0.15 W/cm^2^, blue, 0.2 W/cm^2^, teal, 0.3 W/cm^2^. (**iii**) Cell viability of U87 cancer cells after incubation with unloaded liposomes, Dox-lipo, or Dox-pp lipo under creasing ultrasound intensities. Cell viability was determined 24 h after treatment exposure using a CCK-8 assay (** *p* < 0.01). Image adapted from Wang © Elsevier (2018) Ref. [84]. (**D**) (**i**) Schematic of LNDFm and LNDFp.i formulation, relying on folic acid receptor-dependent uptake for delivery of doxorubicin in P-glycoprotein-positive cancer cells. (**ii**) Uptake of doxorubicin in P gp/FAR negative cells, MDA-MB-231 (drug resistant) cells, and TUBO cells over 72 h while incubated with different treatment types, including LNDFm and LNDFpi (° *p* < 0.01, * *p*/^#^ *p* < 0.001). (**iii**) TUBO xenograft tumor growth in treated with control (ctrl), Dox (D), Caelyx, nitroxy-doxorubicin (ND), liposomal ND (LND), and LNDF, as measured by calipers. (ND/LND/LNDF vs. Ctrl group: * *p* < 0.01; ND/LND/LNDF vs. D: ° *p* < 0.01; LNDF vs. ND: ^#^ *p* < 0.001; LNDF vs. LND: *p* < 0.05.) Image from Gazzano © Elsevier (2018) Ref. [69].

Peptide–liposome bioconjugate systems can often be combined and used with other technologies for a dual-purpose drug delivery system. Park and colleagues established a novel microbubble–liposome complex conjugated to interleukin-4 receptor (IL4R)-targeting peptide ligands for theranostic treatment of brain tumors (Figure 6A) [112]. In this study, DSPE-PEG-PDP liposomes were prepared using thin-film hydration and remote-loaded with DOX via an ammonium sulfate gradient. Subsequently, thiol-active SF_6_ gas-filled microbubbles were covalently conjugated to thiolated DOX-loaded liposomes (MB-Lipo (DOX)). The amine functional group on the DOX-liposome surface was converted to an active maleimide functional group using sulfosuccinimidyl 4-(N-maleimidomethyl)cyclohexane-1-carboxylate (sulfo-SMCC). IL4R-targeting peptides (IL4RTPs) were conjugated to the liposomes by the reaction between maleimide groups on the MB-Lipo (DOX) and thiol groups on the peptides. High performance liquid-chromatography (HPLC) was used to separate MB-Lipo (DOX)-IL4RTPs from unreactive substrates. The researchers hypothesized that the presence of the IL4RTP would improve the affinity of the MB-Lipo (DOX) to IL4R-expressing brain tumor cells (U87MG), and attached microbubbles could be used for ultrasound imaging and directed treatment of brain tumors. When treated with MB-Lipo (DOX)-IL4RTPs and ultrasound at >0.8 W/cm^2^, U87MG cells experienced a 30% decrease in viability as compared to cells treated without the targeting peptide or non-IL4R expressing cells (H460) (Figure 6A). In this way, Kim and colleagues demonstrated the benefit of targeting peptide conjugation to the liposome surface.

In a study by Yang and colleagues, another dual-purpose liposomal system was developed involving the use of two different peptides conjugated to the liposome surface for delivery of therapeutic siRNA [105]. NGR peptides were attached to improve liposomal drug targeting to the site of CD13-positive tumors, while a photolabile-caged, cell-penetrating peptide (pcCPP) was attached to the liposome surface to provide the system with a conditional near-infrared light-depending cell-penetrating functionality (Figure 6B). Briefly, pcCPP and NGF were terminated with cysteine to introduce a free sulfhydryl group and then conjugated to DSPE-PEG2000-Mal and DSPE-PEG5000-Mal respectively by via sulfhydryl-maleimide reaction. Liposomes were prepared by thin film hydration of SPC, cholesterol, DC-cholesterol, and DSPE-PEG2000 (DSPE-PEG5000-NGR for NGR-conjugated liposomes) and hydrated with a siRNA aqueous solution (siRNAs used included si.N.C. (control), c-myc siRNA, and FAM-siRNA). The pcCPP-DSPE-PEG2000 was added using the post-insertion technique. The researchers subsequently showed that 61% of HT-1080 CD13-positive cancer cells underwent apoptosis when treated with near infrared pretreated pcCPP/NGR-LPs containing c-myc siRNA versus 33% of cells undergoing apoptosis after transfection with N-LPs (LPs carrying c-myc siRNA with a control-peptide). Furthermore, in an in vivo study with mice bearing HT-1080 tumor xenografts, the researchers demonstrated therapeutic efficacy with their dual-purpose liposome by showing that mice injected intravenously every other day for 10 days with 1.2 mg/kg siRNA in near infrared-activated pcCPP/NGR-LPs had a significantly smaller tumor volume and weight versus mice treated with free siRNA or simpler liposomes (Figure 6B). On average, mice treated with pcCPP/NGR-LP had an average tumor weight of approximately 0.2 g, versus the second most successful treatment (pcCPP-LP activated with near infrared) with an average tumor weight of approximately 0.4 g; mice treated with free siRNA had an average tumor weight of 1.1 g.

### 4.4. Other Small Molecule–Liposome Bioconjugates

While antibodies, aptamers, and peptides are the most commonly used for liposome targeting, a host of other small molecules have been conjugated to liposomes to improve drug delivery capabilities. These include carbohydrates and glycopolymers, such as hyaluronic acid or galactose, which are known to actively bind to specific cell types [68,113,114,115], and small molecules like porphyrins [84]. Wang and colleagues developed DOX-loaded liposomes consisting of DSPC, DOPE, porphyrins, and DSPE-PEG2000 using the thin film hydration method and remote-loading using an ammonium sulphate gradient [84]. The resulting liposome (pp-lipo) contained porphyrins embedded within the liposome lipid bilayer (Figure 6C). Ultrasound-induced stimulation of the porphyrins within the lipid bilayer caused lipid oxidation, which led to release of encapsulated DOX; the researchers showed that by using an ultrasound intensity of 0.3 W/cm^2^, DOX-pp-lipo released 3× the amount of drug compared to DOX-lipo (Figure 6C). Furthermore, the researchers showed a significant decrease in U87 cancer cell viability in DOX-pp-lipo treated cells versus cells treated with DOX-lipo or no DOX due to cellar uptake of released DOX (Figure 6C). In vivo, the researchers were able to increase survivability in U87-xenograft mice models, and showed a significant reduction of tumor volume by 13 days post-treatment.

The use of vitamins as liposome ligands has also been explored to treat unusual cancer cell types that overexpress vitamin receptors such as folate, rhamnose, or tocopherol [90,116,117]. A recent study exploring the use of folic acid (FA) as a targeting ligand was carried out by Gazzano and colleagues, who utilized FA-targeted liposomes carrying nitrooxy-doxorubicin (ND) for treatment against P-glycoprotein (P-gp) and folate-receptor (FAR) expressing tumors [102]. Two different FA-targeted liposomes were made; liposomal ND, and FA-(LNDF)m, which was produced by adding the FA-PEG-DSPE conjugate to other phospholipids within the thin film hydration technique, while ND was added during liposome synthesis. Alternatively, LNDFpi was made by adding FA-PEG-DSPC to preformed DSPC/Chol/mPEG-DSPE liposomes containing ND using the post-insertion method (Figure 6D). The researchers were able to demonstrate specificity within their drug delivery platform, observing approximately 100 nmol/mg cell protein in P-gp/FAR positive cell lines (MDA-231 and TUBO) as opposed to 50 nmol/mg cell protein in the P-gp/FAR negative cell line MCF-7 (Figure 6D) after 72 h. Within a mouse TUBO-cell xenograft model, the researchers also observed that treatment with the LNDF liposomes resulted in a tumor volume of 1200 mm^3^ over 21 days, while mice treated with LND (without folic acid) experienced an average tumor volume of 3000 mm^3^. Mice treated with non-liposomal ND had tumors with an average volume of 4000 mm^3^ (Figure 6D).

## 5. Clinical Use of Liposomes: Current State of the Art

The first liposomal product to be approved by the FDA was Doxil^®^, a liposomal formulation of doxorubicin first used in 1995 for treatment of ovarian cancer and AIDS-related Kaposi’s Sarcoma [118]. Today, over 12 liposomal formulations have obtained regulatory approval for clinical use for the treatment of bacterial infection, cancer, and fungal disease [119,120], and eye, skin, and respiratory disorders [90]. Liposomal formulations are now among the most successful of the nanomaterial-based therapeutics currently approved for use in the clinic [119,121], and approximately 330 clinical trials around the world have begun since the beginning of 2015 and are either recruiting or still ongoing (without suspension), according to a search in “clinicaltrials.gov”. Meanwhile, approximately 100 clinical studies have been completed within this timeframe. A representative listing of ongoing and recently completed clinical trials can be found in Table 2.

Since 2015, three new liposomal formulations have been approved for clinical use by the FDA: Onivyde^®^ (2015), Vyxeos^®^ (2017), and Onpattro^®^ (2018). Onivyde^®^ is a PEGylated nanoliposomal form of irinotecan that has been approved for use in combination with gemcitabine in treating advanced pancreatic cancer patients but also shows promising use in therapy for other solid tumor types [122]. Irinotecan is encapsulated in Onivyde^®^ via a modified gradient-loading method utilizing sucrose octasulfate [122]. Currently, Onivyde^®^ is being investigated for other uses in additional clinical trials, including a Phase III trial for use in treatment of small cell lung cancer in conjunction with topotecan (clinicaltrials.gov; NCT03088813). Onivyde^®^ has multiple pharmacokinetic benefits over conventional irinotecan, including increased drug encapsulation and loading efficiency, protection of the drug in its active configuration, prolonged circulation time, and sustained release, and reduced host toxicity and contact with the gastrointestinal tract [122]. Although there are significant advantages to the use of Onivyde^®^ over conventional irinotecan, indications of drug toxicity seen during Phase III trials included neutropenia, fatigue, diarrhea, and vomiting [122]. Vyxeos^®^ (CPX-351) is a liposomal form of daunorubicin and cytarabine (1:5 molar ratio; liposome composed of 7:2:1 DSPC, DSPG, cholesterol). It has been approved for treatment of acute myeloid leukemia. Cytarabine is encapsulated by mixing cytarabine solution and liposomes in copper-free formulation buffers, and incubating the mixture past phase transition temperature. Daunorubicin can be encapsulated post-liposome formation by dissolving in ethylenediaminetetraacetic acid (EDTA) buffer at neutral pH, then incubating with cytarabine-loaded liposomes [123]. Patients in clinical trials receiving Vyxeos^®^ had an overall median survival of 9.63 months versus 5.59 months on conventional treatment, due to increased efficacy from the synergistic effect of the coencapsulated drugs [119,124]. However, despite these benefits, notable toxicity was found in some patients during phase III trials (>20% incidence) including febrile neutropenia, bacteremia, and pneumonia, and treatment had to be discontinued in 18% of recipients due to side effects; despite this, the overall safety profile was similar to conventional chemotherapy [124].

The most recently approved liposomal therapy is Onpattro^®^, which is also novel due to its use of antitransthyretin siRNA (patisiran) as a therapeutic agent. Onpattro^®^ is currently approved to treat transthyretin-mediated (ATTR) amyloidosis. The liposomal portion of Onpattro^®^ is composed of ionizable cationic lipids (DLin-MC3-DMA), phospholipid (DSPC), cholesterol, and PEG2000-C-DMG, combined via rapid mixing under acidic pH. The drug is encapsulated as pH neutralizes, whereby smaller liposomes fuse into a large lipid nanoparticle [125]. In the body, the liposome is directed to the endosome, where DLin-MC3-DMA becomes cationic due to the low pH. Following this localization to the endosome, osmotic swelling occurs within the endosome, resulting in until endosomal rupture and allowing the encapsulated siRNA to reach the cytosol. There, the siRNA inhibits synthesis of the transthyretin protein, decreasing its levels in serum and tissue deposits [125]. During Phase III clinical trials, Onpattro^®^ showed an 81% reduction in transthyretin production and improved muscle strength, sensation, reflexes, and heart rate compared to patients treated with a placebo [118,126]. However, approximately 20% of patients had mild to moderate side effects including peripheral edema and infusion related reactions (diarrhea, nausea, dizziness, etc.), although the frequency of severe adverse effects was comparable to the placebo group (28% in the Onpattro^®^ group and 36% in the placebo group).

Presently, clinical trials for liposomal products are predominantly geared toward improving already-approved drug formulations by encapsulation within the liposome. In particular, the exploration of liposomal formulations of chemotherapeutics is popular as the liposome is able to shield the body from the cytotoxic effects of the encapsulated drug. For example, Lipocurc^TM^ is a liposomal form of curcumin in Phase I/II clinical trials for patients with advanced cancer that had failed standard of care therapy (clinicaltrials.gov; NCT02138955). Within the study, no dose limiting toxicity was observed at doses of under 300 mg/m^2^, although adverse effects occurred in the majority of patients and disease progression continued in almost all patients [127]. Another chemotherapeutic formulation currently in active clinical trials is liposomal annamycin, for the treatment of acute myeloid leukemia (clinicaltrials.gov; NCT03315039).

With the success of Onpattro^®^, siRNA-loaded liposomes are also of interest as a new therapeutic method. There are currently multiple siRNA-based lipid therapeutics in clinical trials, for treatments of diseases ranging from advanced cancers to chronic Hepatitis B [128]. One example of a current siRNA-based liposome drug in Phase I/II clinical trials for treatment of advanced pancreatic carcinoma and solid tumors is AtuPLEX^TM^. AtuPLEX^TM^ is a cationic liposome encapsulating siRNA that targets Protein Kinase N3; loading can be done during synthesis with pH sensitive lipids or mixing to create lipoplexes [129]. During Phase I trials, the drug showed low (1–2) grade toxicity and produced a stable disease state in 41% of patients within 8 weeks. Phase I/II trials performed in conjunction with gemcitabine and found patients treated twice weekly with AtuPLEX^TM^ showed improvement in health status [130].

Overall, there are various established benefits of the use of a liposomal encapsulation compared to treatment with conventional formulations; particularly improved delivery to the tumor site, efficacy, and decreased systemic toxicity over conventional formulation, which is a typical goal for most new drugs advancing to clinical trials [119]. While toxicity is typically noted within clinical trials, liposomal formulations have a common theme of reducing the toxicity of the encapsulated drug [119].

**Table 2:** Liposome-based drug therapies in clinical trials or recently approved for use.

Drug Name	Year	Drug Cargo	Application	Trial Phase	ClinicalTrial.Gov ID or [ref]
Alprostadil	2019 (2021)	Alprostadil	Peripheral artery disease	Phase II	NCT04197323
Amikacin	2019	Amikacin (antibiotic)	Mycobacterium abscesses lung disease	Phase II	NCT03038178
Annamycin	2018 (2021)	Annamycin	Acute myeloid leukemia	Phase I	NCT03315039
ARB-001467 TKM-HPV	2018	Three siRNA targeting HBV RNA	Hepatitis B Virus	Phase II	NCT02631096, [128]
Atu027	2016	Atu027 (siRNA) targeting PKN3 (in conjunction with Gemcitabine)	Advanced pancreatic carcinoma	Phase I/II	NCT01808638, [130]
Bupivacaine	2018 (2021)	Bupivacaine	Pain control during colorectal surgery	Phase III	NCT03702621
Cyclosporine A	2019 (2022)	Cyclosporine A	Bronchiolitis Obliterans, Lung Transplant Rejection	Phase III	NCT03657342 NCT03656926
E7389	2017 (2021)	E7389	Solid tumor therapy (breast cancer, adenoid cystic carcinoma, gastric cancer, esophageal cancer, and small cell lung cancer)	Phase I	NCT03207672, [131]
FF-10832 Gemcitabine	2018 (2021)	Gemcitabine (in conjunction with free Paclitaxel)	Advanced solid tumors	Phase I	NCT03440450
HIV-1 gp41 MPER-656	2019 (2021)	HIV-1 gp41	HIV-1 vaccine	Phase I	NCT03934541
LipocurcTM	2017	Curcumin	Advanced cancer (solid tumors) who have failed standard of care therapy	Phase I/II	NCT02138955, [132]
ND-L02-s0201	2016	Heat shock protein 47 siRNA	Hepatic fibrosis	Phase I	NCT02227459, [128]
Onivyde^®^	2015	Irinotecan, Fluorouracil	Metastatic pancreatic cancer	FDA approved	[121]
Onpattro^®^	2018	siRNA (antitransthyretin)/Patrisiran	Transthyretin-mediated amyloidosis	FDA approved	[118,125]
ThermoDox^®^ (Tardox)	2019 (Ph I), 2018 (Ph III)	DOX	Temperature-triggered DOX release; liver cancer (Ph I), hepatocellular carcinoma (Ph III)	Phase I & Phase III	[120,133], NCT02181075 (Ph I) NCT02112656 (Ph III)
TLC599	2019 (2021)	Dexamethasone	Knee osteoarthritis	Phase III	NCT04123561
Vyxeos^®^	2017	Daunorubicin and Cytarabine	Acute myeloid leukemia	FDA approved	[118,119,121]

Abbreviations: DOX, doxorubicin; E7389, eribulin; HBV, Hepatitis B Virus; HIV-1 gp41, Human Immunodeficiency Virus-1 glycoprotein 41; MPER, membrane-proximal external region; PEG, polyethylene glycol; PKN3, protein kinase N3.

## 6. Conclusions

Advances in harnessing bioconjugation strategies for drug incorporation into liposomes and specific targeting of liposomes to points of interest has exploded in recent years and brings with it much promise for the future of liposomes for drug delivery. We expect many more liposome carriers to enter clinical trials and become approved for clinical use in the next few years. For example, two promising vaccine candidates for the COVID-19 pandemic from Pfizer (clinicaltrials.gov; NCT04368728) and Moderna (clinicaltrials.gov; NCT04405076) rely on liposomes as the vehicle for RNA delivery [134]. Looking forward, based on the success of the recently approved liposomal formulations Onivyde^®^, Vyxeos^®^, and Onpattro^®^, we anticipate that combination approaches using multiple bioconjugation strategies to achieve complex liposome designs with multiple, theranostic and imaging uses will become even more prevalent. As discussed in Section 5, formulations currently in clinical trials are using the liposome and various bioconjugation strategies not only to shield the body from unwanted drug leakage, but also to improve the efficacy of drugs like chemotherapeutics and antibiotics. Specifically, by improving the effects of antibiotics and reducing their off-target effects, we can repurpose drugs and fight growing antibiotic resistance. Given the recent improvements in these areas, the natural next step of clinically relevant liposome bioconjugates would be for multimodal, theranostic treatment of cancer and disease. Thus, it is imperative that researchers continue to investigate the effects of these bioconjugation strategies on cellular behavior, and the effects of cellular environments on the liposome carriers to best design liposomes for particular applications.
